# The Origin and Initial Rise of Pelagic Cephalopods in the Ordovician

**DOI:** 10.1371/journal.pone.0007262

**Published:** 2009-09-30

**Authors:** Björn Kröger, Thomas Servais, Yunbai Zhang

**Affiliations:** 1 Museum für Naturkunde, Leibniz Institute for Research on Evolution and Biodiversity at the Humboldt University Berlin, Berlin, Germany; 2 Université de Lille 1, UMR 8157 du CNRS Géosystémes, Villeneuve d' Ascq, France; 3 Nanjing Institute of Geology and Paleontology, Nanjing, China; Smithsonian Institution, National Museum of Natural History, United States of America

## Abstract

**Background:**

During the Ordovician the global diversity increased dramatically at family, genus and species levels. Partially the diversification is explained by an increased nutrient, and phytoplankton availability in the open water. Cephalopods are among the top predators of todays open oceans. Their Ordovician occurrences, diversity evolution and abundance pattern potentially provides information on the evolution of the pelagic food chain.

**Methodology/Principal Findings:**

We reconstructed the cephalopod departure from originally exclusively neritic habitats into the pelagic zone by the compilation of occurrence data in offshore paleoenvironments from the Paleobiology Database, and from own data, by evidence of the functional morphology, and the taphonomy of selected cephalopod faunas. The occurrence data show, that cephalopod associations in offshore depositional settings and black shales are characterized by a specific composition, often dominated by orthocerids and lituitids. The siphuncle and conch form of these cephalopods indicate a dominant lifestyle as pelagic, vertical migrants. The frequency distribution of conch sizes and the pattern of epibionts indicate an autochthonous origin of the majority of orthocerid and lituitid shells. The consistent concentration of these cephalopods in deep subtidal sediments, starting from the middle Tremadocian indicates the occupation of the pelagic zone early in the Early Ordovician and a subsequent diversification which peaked during the Darriwilian.

**Conclusions/Significance:**

The exploitation of the pelagic realm started synchronously in several independent invertebrate clades during the latest Cambrian to Middle Ordovician. The initial rise and diversification of pelagic cephalopods during the Early and Middle Ordovician indicates the establishment of a pelagic food chain sustainable enough for the development of a diverse fauna of large predators. The earliest pelagic cephalopods were slowly swimming vertical migrants. The appearance and early diversification of pelagic cephalopods is interpreted as a consequence of the increased food availability in the open water since the latest Cambrian.

## Introduction

Cephalopods are swimming animals and as such often considered as organisms of the free water column. Cephalopods of today inhabit nearly the complete spectrum of marine environments, they live in rocky intertidal zones, in the blue ocean, related to the sea bottom and fully pelagic. Their widespread habitats are accompanied by a wide variety of life habits.

Early Paleozoic cephalopods differ drastically from their modern relatives, and initially a global distribution in a wide variety of paleoenvironments did not exist. The earliest cephalopods appeared in the latest Cambrian in North China, by then a shallow carbonate platform in tropical low latitudes. Cephalopods diversified rapidly in the latest Cambrian but were confined to neritic habitats of low paleo-latitudes up to the middle Early Ordovician [1]. These early cephalopod occurrences are often found in the vicinity of thrombolitic buildups, and associated with gastropods and other mollusks, a facies, called “cephalopod facies” by some authors [2]. During the Ordovician the level of ecosystem complexity increased globally strongly and organismal ecospace utilization intensified significantly [3,4]. The expansion of cephalopod habitats and life habits into more open water paleoenvironments and higher latitudes during this exceptional time interval was never comprehensively investigated and reviewed. The data are widely dispersed in the literature, and in paleobiological databases, and are difficult to interpret.

Here, we reconstruct the cephalopod departure into the pelagic realm by the compilation of occurrence data in offshore paleoenvironments from the Paleobiology Database (PBDB), and from own data, by evidence of the functional morphology of cephalopods and the taphonomy of selected faunas.

## Results

### Late Cambrian–Early Ordovician cephalopods in offshore depositional settings

Late Cambrian and early Tremadocian cephalopod occurrences are reported from off-shore shelf carbonates from South China, which are interpreted as deposited below normal storm wave base in some cases, but clearly represent neritic habitats [5], [6]. Late Cambrian cephalopod occurrences in deeper water settings, representing depositional depths below the neritic zone, are not known. In the Early Ordovician they are rare: of the 70 Early Ordovician deep subtidal sediment, basinal and black shale collections in the Paleobiology Database only 3 ( = 4%) contain cephalopods (see Appendix S1). No Late Cambrian and Early Ordovician cephalopods are known from black shales in basinal settings (equivalent to Benthic Association 6, BA6 [7]).

The oldest cephalopods unequivocally known from deeper water depositional environments off the carbonate platforms are mid Tremadocian (IGCP 410 time slice 1b [8]) in age and of high latitude paleogeographical provenance. Mid Tremadocian in age is the small, orthoconic cephalopod *Slemmestadoceras attavus*, which rarely occurs in a black nodule bed in the Bjørkåsholmen Formation (*Paltodus deltifer* Conodont Zone) of the Oslo Region, Norway. The bed is considered as being deposited well below the normal wave base or even below the storm wave base [9]. The second and only other known mid Tremadocian cephalopod occurrence outside the tropical shallow water “cephalopod facies” is a thin cephalopod limestone (bed Tu-35.9 [10]) in the dark shales of the Rio Salinas Member, Tiñu Formation (*Paltodus deltifer* Conodont Zone), Oaxaca, Mexico. The bed consists of masses of, often telescoped, orthoconic cephalopods, predominantly *Rioceras*, and is interpreted as a tempestite [10]. The cephalopods of the Bjørkåsholmen and Tiñu Formation with their orthocerid conch morphologies are unusual for the time. Among them are the potentially earliest representatives the Orthocerida, cephalopods which are more common and characteristic in the Middle Ordovician and later in the Paleozoic.

**Figure 1:**
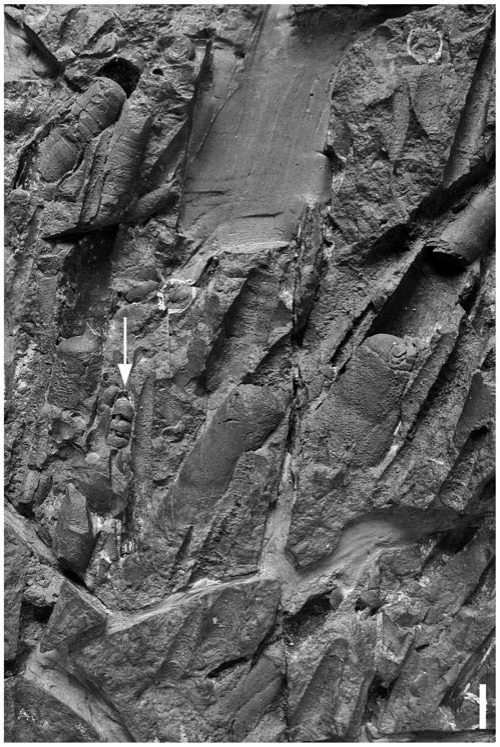
Nodule with masses of orthoconic nautiloids from La Maurerie Formation, earliest Floian, Montagne Noire, France. Arrow highlights *Bactroceras*. Scale bar equals 1 cm.

The only late Tremadocian cephalopod occurrence known from deeper water settings is the yet undescribed association from nodules in the upper parts of the black shales of the Saint Chinian and the lower La Maurerie Formation (IGCP 410 time slices 1b–2a ), Montagne Noire, France [11]. The fauna consists exclusively of orthocones, among them a few orthocerid-like forms [12], including the orthocerid *Bactroceras* (Figure 1).

Floian deep offshore sediments with cephalopods are known from Bolivia [13] and Wales [12], only. Both associations are dominated by orthocones. The fauna from Wales contains a number of remarkable stem group orthocerids such as *Polymeres*, and *Semiannuloceras* [12], [14]. *Bactroceras* is the earliest known orthocerid, and *Polymeres*, *Semiannuloceras* are other early representatives of the Orthocerida. The Orthocerida are the oldest neocephalopods [15], which comprise all modern cephalopods, except *Nautilus*. Therefore, the key for the reconstruction of the neocephalopod origin is in these Tremadocian and early Floian offshore occurrences.

**Figure 2:**
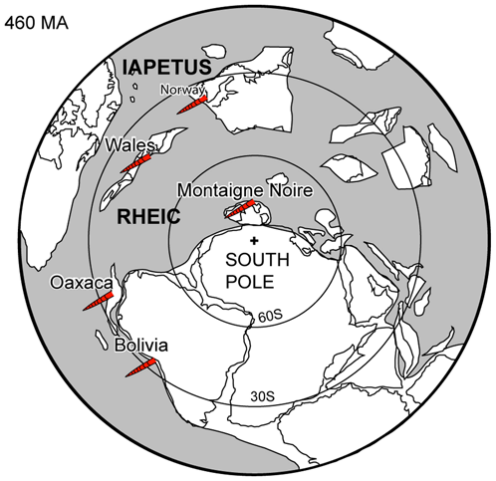
Early Ordovician cephalopod occurrences in distal and deep marine depositional settings. Map simplified from [97].

In conclusion the first cephalopod associations in deep basinal settings occur in the Early Ordovician. These occurrences are rare, and concentrated in high paleolatitudes (Figure 2). The offshore associations are dominated by orthoconic ellesmerocerids, orthocerids, and orthocerid like orthocones. Breviconic and coiled forms are absent or extremely rare. With this, the the Early Ordovician offshore associations differ considerably from their shallow water equivalents.

### Middle Ordovician cephalopods in offshore depositional settings

Of the 94 Middle Ordovician collections in the Paleobiology Database from deep subtidal, basinal settings, and black shales 11 collections (12%) contain cephalopods. With our additional compilation from the cephalopod literature a total of 37 Middle Ordovician collections with nautiloids (see Appendix S1) is known. Most common in these collections are orthocerids; 76% contain orthocerids, followed by large orthoconic endocerids (51% of the collections), and lituitids (27% of the collections). Only four collections (11%) contain breviconic oncocerids and ellesmerocerids.

Only two cephalopod records are known from Dapingian collections with depositional depths below 200 m ( = below the neritic zone) or from black shales: the record of *Bactroceras* from the Pontyfenni Formation (*Isograptus gibberulus* Graptolite Zone) from Wales, UK [12], and a “*Geisonoceras*” from black shales of the Chikunsan beds of North Korea [16]. The depositional depth of the Pontyfenni Formation is estimated as below 300 m [17].

In contrast Darriwilian cephalopod occurrences in deep subtidal and basinal settings are widespread and known from Canada, China, the Czech Republic, Norway, North Korea, the UK, Sweden, and Spain (see Appendix S1). Some better known collections are: (1) The cephalopod association of the Aber Mawr Shale Formation (*Didymograptus artus* Graptolite Zone), Darriwilian, of Wales which was revised by Evans [12]. The Aber Mawr Shale Formation comprises dark mudstones and shales intercalated with rhyolitic allochthonous tuff horizons with an estimated depositional depth of below 300 m [17]. In the Aber Mawr Shale Formation the slender, orthoconic ellesmerocerids *Sacerdoceras*, and *Bathmoceras*, the orthocerid “*Orthoceras*” *avelinii*, and the orthocerid-like *Cyclorangeroceras* occur. (2) The Šárka Formation, Darriwilian, Prague Basin, consists of dark shales with fossil bearing nodules. The *Euorthisinia* (brachiopod)-*Placoparia* (trilobite) Community [18] of the Šárka has an estimate depositional depth of clearly below normal wave base (BA3–4) and yields 21 cephalopod species, of predominantly orthocerids [19]. *Bactroceras*, and *Bathmoceras* are remarkable as recurring elements. Orthoconic nautiloids occur in a comparatively fossil poor deep water interval of the lower Šárka Formation with dominant pelagic organisms such as graptoloids and phyllocarids [20]. (3) The Kuniutan Formation, Darriwilian, Yangtze Gorge area, China, consists of a nodular purplish wacke-mudstone. The formation is interpreted as representing an outer shelf depositional environment with estimated water depths of 220–340 m [21], [22]. The cephalopods in the Kuniutan are dominated in abundance by the endocerid *Dideroceras*, orthocerids, and the lituitids *Ancistroceras* and *Sinoceras*.

**Figure 3:**
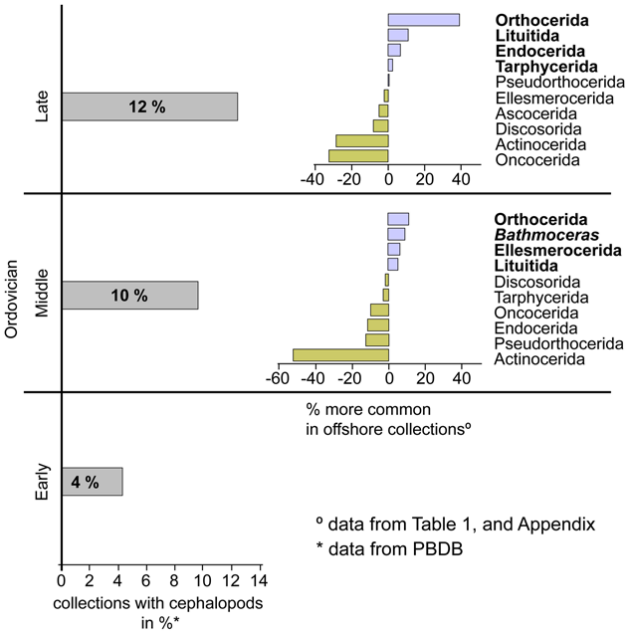
Ordovician cephalopod occurrences in deep/distal/black shale depositional settings and comparison with shallow settings.

These offshore occurrences strongly contrast with cephalopod associations of shallower settings. For example, actinocerids in abundance strongly dominate the shallower depositional settings of the North China Platform whilst lituitids and orthocerids dominate the deeper settings [21]. Our own data show that orthocerids are also the most common cephalopods in Middle Ordovician shallow water settings. But in contrast to collections from deeper water sediments, the diversity at higher taxonomic levels is much higher in shallow water settings, and there the breviconic oncocerids and discocerids are clearly more common and diverse. Globally, orthocerids (65% of collections), endocerids (63% of collections) and actinocerids (52% of collections) are most common in shallow subtidal, reefal and peritidal settings (Figure 3, Table 1)

**Table 1:** Comparison of relative occurrences of selected cephalopod taxa in shallow, and deep/distal/black shale depositional settings.

	occurrences in % of cephalopod collections+		
	taxon	neritic zone	deep, distal, and black shale marine
**Middle Ordovician**			
	Actinocerida	52	0
	*Bathmoceras*	2	11
	Discosorida	2	0
	Ellesmerocerida*	2	8
	Endocerida	63	51
	Lituitida	21	27
	Oncocerida	12	3
	Orthocerida	64	76
	Pseudorthocerida°	12	0
	Tarphycerida	19	16
**Late Ordovician**			
	Actinocerida	39	10
	Ascocerida	12	6
	Ellesmerocerida	2	0
	Endocerida	8	14
	Discosorida	12	4
	Lituitida	1	11
	Oncocerida	59	27
	Orthocerida	21	60
	Pseudorthocerida°	15	16
	Tarphycerida	15	17

+ see Appendix S1 for data.

* *Bathmoceras* exclusive.

° using the classification criteria of [98].

Conclusively, it is evident that cephalopod associations occur worldwide in the Middle Ordovician offshore settings. Slender, orthocones such as orthocerids and orthoconic endocerids are most abundant. The high abundance of lituitids and the rarity of actinocerids, oncocerids and other breviconic forms is remarkable.

### Late Ordovician cephalopods in offshore depositional settings

Of the 458 collections in the Paleobiology Database from Late Ordovician deep subtidal, and basinal settings, and black shales 57 collections ( = 12%) contain cephalopods. Our additional compilation from the cephalopod literature results in a total of 186 Late Ordovician collections with cephalopods from deep settings and black shales (see Appendix S1). Most common in these collections are orthocerids, (60% of the collections), oncocerids (27% of the collections), and tarphycerids (17% of the collections).

**Figure 4:**
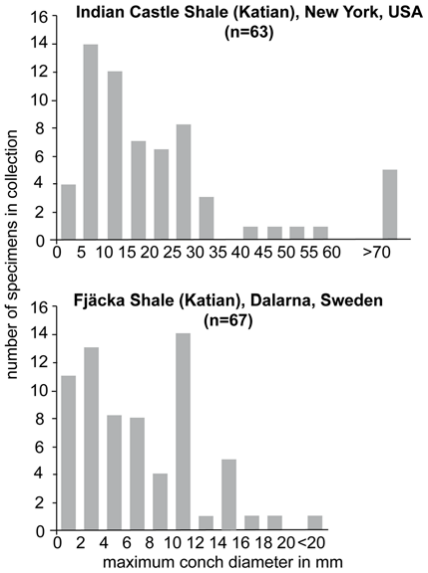
Frequency distribution of maximum conch diameter of orthoconic cephalopods in Late Ordovician black shales.

Late Ordovician cephalopod associations from deep subtidal and basinal depositional environments and black shales occur worldwide (see Appendix S1). We analysed three examples, two of them are from black shales with an abundance, and diversity restricted benthic fauna: (1) The Indian Castle (Utica) Shale, (uppermost *Orthograptus ruedemanni* – *Climagraptus pygmaeus* Graptolite Zone), late Katian, New York comprises dark, laminated, slightly calcareous clay shales with abundant graptolites, and small inarticulates [23]. The fauna of the shales was described in detail and is interpreted as BA 6, representing a basinal dysoxic – anoxic, aphotic – dysphotic depositional environment; cephalopods are comparatively common [23], [24]. Several species of “*Geisonoceras*” were described from the Indian Castle Shale which must be correctly assigned to *Isorthoceras*, and in the case of “*G. amplicameratum*” to *Ordogeisonoceras*, large endocerids, two species of *Trocholites*, and an *Oncoceras* [24], [25]. The analysis of a large collection of Utica slabs in the New York State Museum (NYSM), Albany, New York, revealed a strong dominance in abundance of *Isorthoceras* in the samples. The 98 cephalopod specimens from the Indian Castle in the NYSM contain 70 orthoconic specimens of predominantly *Isorthoceras*, 17 *Trocholites*, 8 *Oncoceras*, and three large fragments of putative endocerids. The frequency distribution of the fragment conch diameter of the orthocerids is roughly unimodal with most fragments of maximum diameter 5–30 mm, which corresponds to a conch length of less than 80 mm (see Figure 4). (2) The Fjäcka Shale, (late *Pleurograptus linearis* Graptolite Zone), late Katian, Dalarna, Sweden is a highly fossiliferous black bituminous shale. The fauna of the Fjäcka Shale is of low diversity, dominated by trilobites, ostracodes and phosphatic brachiopods, and interpreted as representing dysoxic bottom conditions [26]. Collections of Fjäcka Shale slabs at the Naturhistoriska Riksmuseet Stockholm (NRM), Sweden, and the Evolutionsmuseet Uppsala (PMU), Sweden contain a total of 78 cephalopods, 91% of them are orthocerids, predominantly *Isorthoceras*. Beside orthocerids the association contains 5 tarphycerids (*Discoceras*), one oncocerid (*Beloitoceras*), and one lituitid (*Tyrioceras*). The frequency distribution of the maximum fragment diameter indicates an average conch length of the *Isorthoceras* specimens of 80–90 mm. (3) The cephalopod association of the Pagoda Formation, mid Sandbian–early Katian, South China is well known [21], [27]. The Pagoda Formation consists of light grey bioclastic micritic mud–wackestones. The depositional depth of the Pagoda Limestone was controversially disputed but most recent investigations assume a deep water setting [22] which is consistent with the depth estimations of 300–400 m based on cephalopod implosion depths [21]. The cephalopods of the Pagoda Formation are strongly dominated by *Sinoceras*, *Michelinoceras* and *Eosomichelinoceras*, with *Sinoceras* representing the predominant genus. *Bactroceras* is mentioned and actinocerids are rare [14], [27].

These three examples of Late Ordovician cephalopod occurrences in black shales and deep water settings are characteristic in being strongly dominated by orthocerids and partly lituitids with a concomitant fauna of endocerids, oncocerids and tarphycerids. The dominance of orthocerids and lituitids in individual collections supports the overall impression of the high abundance of these cephalopods in Late Ordovician deep water settings and black shales. The offshore cephalopod occurrences strongly contrast with associations from shallower settings. Our own data show that oncocerids dominate Late Ordovician cephalopod faunas from shallow water settings (60% of the collections contain oncocerids), followed by actinocerids (39% of the collections) (Table 1). Orthocerids occur only in 21% of the shallow water collections, and lituitids, which are typical and especially common in offshore settings of the Yangtze Platform are very rare (Figure 2).

## Discussion

The morphology and the taphonomic features of the cephalopod occurrences in deeper depositional settings, and in black shales give additional evidence for their original life habit and habitat.

### Morphological evidence for vertical movement, and pelagic habitats

#### Apex morphology

The apex of the cephalopod shell represents the earliest growth stages. It provides data on the egg-, and hatchling size, and of the potential hatchling life style. Our analysis of the occurrence data indicates a disproportionately high abundance of orthocerids and lituitids in the Ordovician black shales and deep water sediments (Figure 3). One striking commonality of orthocerids and lituitids is the spherical, comparatively small conch apex [28].

The apex of *Bactroceras* is sub-spherical and measures about 1 mm in diameter, it was repeatedly found in sediments from deep subtidal depositional settings [12], [29]. The apex of *Orthoceras* and most other Early Ordovician orthocerid-like orthocones is not known. But the spherical apices of all known Middle Ordovician orthocerids and lituitids allows the conclusion that the apex of the Orthocerida and the Lituitida is generally spherical [28], [30]. In contrast the apex is large and conical in all other Ordovician nautiloids (e.g. [31], [28], [32] and references therein).

**Figure 5:**
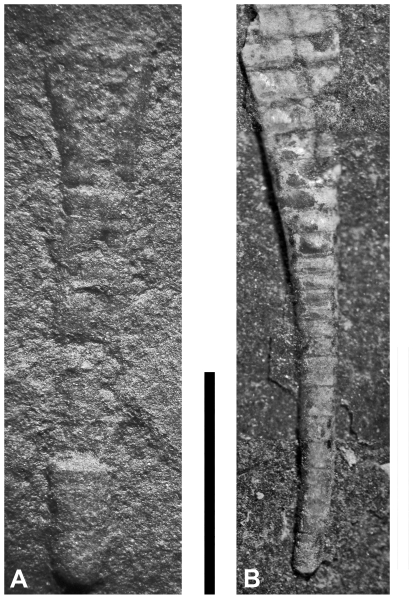
Apices of orthoconic cephalopods from Late Ordovician black shales. A. ?*Isorthoceras* sp., NYSM 17619 from Indian Castle Shale, late Katian, New York. B. Orthocerida indet., NRM-PZ 8874 from Fjäcka Shale, late Katian, Dalarna, Sweden. Scale bar equals 5 mm.

Orthocerid apices are often preserved in Late Ordovician black shales [33, pl. 8, figs 3–10] (Figure 5). These apices are the smallest known from the Ordovician, comprising diameters of the first spherical chamber of about 0.5 mm, only. The small protoconch size and the voluminous, potentially gas filled, first chamber greatly enhanced the buoyancy and swimming ability of the eggs and the cephalopod hatchlings. Additionally, a small protoconch size can be interpreted as evidence for a small yolk mass in the eggs, and probably an early planktonic feeding habit of the hatchlings, comparable with planktotrophic gastropod larvae [34]. The occurrence of planktotrophic gastropod larval shells, and bactritoid embryos and hatchlings is reported from the oxygen depleted Early Carboniferous Ruddle Shale [35]. The Ruddle Shale contains common bactritoids, Pseudorthocerida and other nautiloids, but only bactritoids with their small spherical protoconchs occur as embryonic shells and early post-hatchlings in the shale. A similar situation can be found in the Late Ordovician Fjäcka Shale, Dalarna, Sweden, here an unusual frequency peak of small orthoconic shells with maximum conch cross section of less than 5 mm occurs (Figure 4). The apices of these orthocones probably belong to *Isorthoceras* and have initial chamber diameters of 0.4–0.5 mm (Figure 5).

#### Colour marks

**Figure 6:**
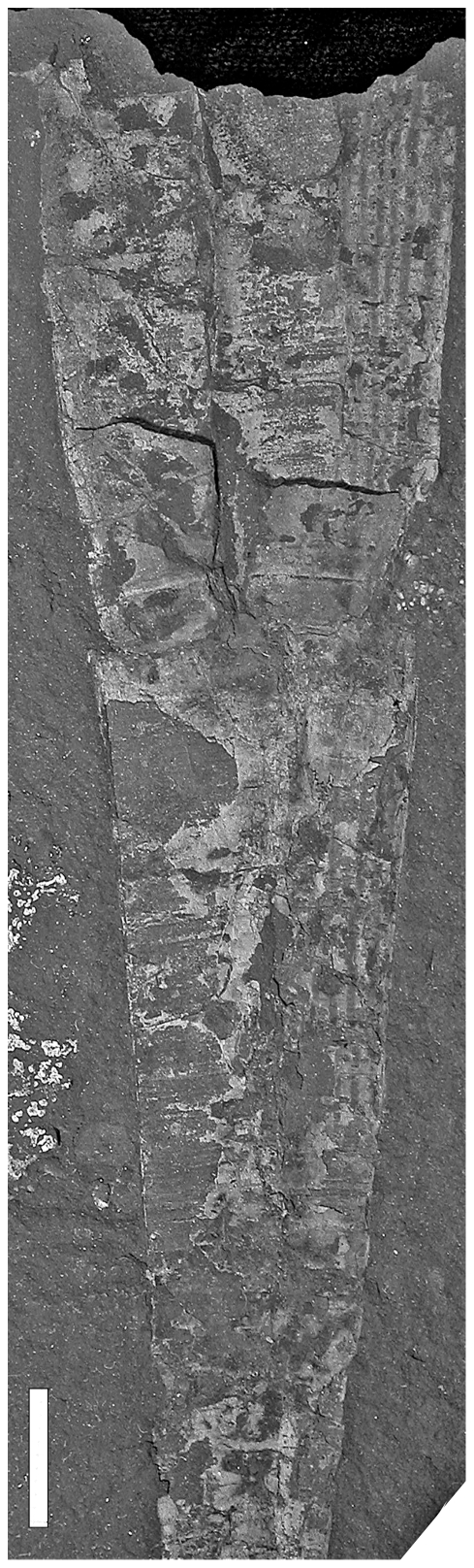
*Isorthoceras tenuitextum* with dorsal color marks. Specimen NYSM 17625 from Indian Castle Shale, late Katian, New York. Scale bar equals 1 cm.

The few cases in which colour marks are preserved on fossil cephalopod conchs provide important information on the life habit. Colour marks are known from *Isorthoceras* (Figure 6). The colour marks are broad longitudinal bands that are confined to one side of the conch. Ruedemann [36] interpreted the colour marked side of *I. tenuitextum* as dorsal, based on material from the Trenton Limestones of New York, USA. The figures of *Orthoceras romingeri*, which is considered to belong to *Isorthoceras* (based on the general shell morphology and the shape of the siphuncle and septal necks), clearly support this interpretation. In *I. romingeri* the longitudinal colour bands are restricted to the concave, dorsal side of the conch [37, fig. 6A]. The presence of colour marks can be interpreted as evidence for an, at least partially photic-zone habitat of *Isorthoceras*. Therefore, colour marks in *Isorthoceras* from the Utica Shale, which is interpreted as deposited under aphotic-dysphotic conditions [23], suggest a life in the free water column. The dorsal position of the colour marks of *Isorthoceras* contrasts with the ventral position of colour marks in fossil cephalopods found in shallower habitats [38]. The ventral colour pattern faced down during the life of the animal, providing a camouflage of the swimming animal from potential bottom dwellers. In contrast a colour pattern confined to the dorsal, upward facing, side of the animal provided a camouflage from freely swimming animals.

#### Siphuncular morphology

The siphuncle is the buoyancy regulation organ of the cephalopods. The siphuncle shape and structure allows to infer the effectiveness and tempo of buoyancy regulation. In lituitids and orthocerids such as *Bactroceras*, *Cochlioceras*, *Orthoceras*, and *Sinoceras* the siphuncle was comparatively thin and tubular with largely suppressed endosiphuncular deposits, and the inner layer of the connecting ring was calcified [39], [40]. The thin tubular and calcified connecting rings secured a maximum mechanical strength against hydrostatic pressure and potentially allowed the migration in great depths [39], [41].

Kröger [42] distinguished between euorthocones and angustocones in orthoconic cephalopods. Euorthocones are characterized by expanded siphuncles with massive endosiphuncular and cameral deposits and angustocones by thin, tubular siphuncles with largely suppressed endosiphuncular deposits. Only angustocones with their narrow siphuncles, often widely spaced, and strong septa had the potential for migrating in great depths. Additionally, angustocones are interpreted as cephalopods with low energy needs [42]. In contrast euorthocones could not withstand high hydrostatic pressures, there large siphuncular surfaces allowed for quick buoyancy changes, but were clearly less energy efficient. This interpretation of the morphological features is supported by the cephalopod occurrences in the cephalopod rich Lower Devonian strata of Morocco, where angustocones are clearly more common and sometimes exceedingly dominate the deeper water or less oxygenated sediments. The Ordovician occurrences show a similar pattern with angustocones concentrated in deep distal, and black shale sediments.

The ability for rapid liquid transport throughout the connecting ring, and therefore for quick buoyancy changes was enhanced in lituitids, orthocerids, and actinocerids by a unique system of fine pores that traversed the connecting ring [40]. This combination of characters enhancing the ability to migrate in great depths, and the ability of buoyancy change in lituitids, and orthocerids supports the interpretation of these forms as cephalopods with low energy needs, which lived as vertical migrants in the free water column.

A peculiar lituitid feature is the occurrence of heavy cameral deposits which cover the complete septal necks and form a characteristic longitudinal lamella [43]. The origin and functional significance of these deposits is disputed controversially (e.g. [19], [44], [45]). Therefore, an evaluation of the life habit of lituitids is difficult at the time and avoided herein.

#### Conch morphology

**Figure 7:**
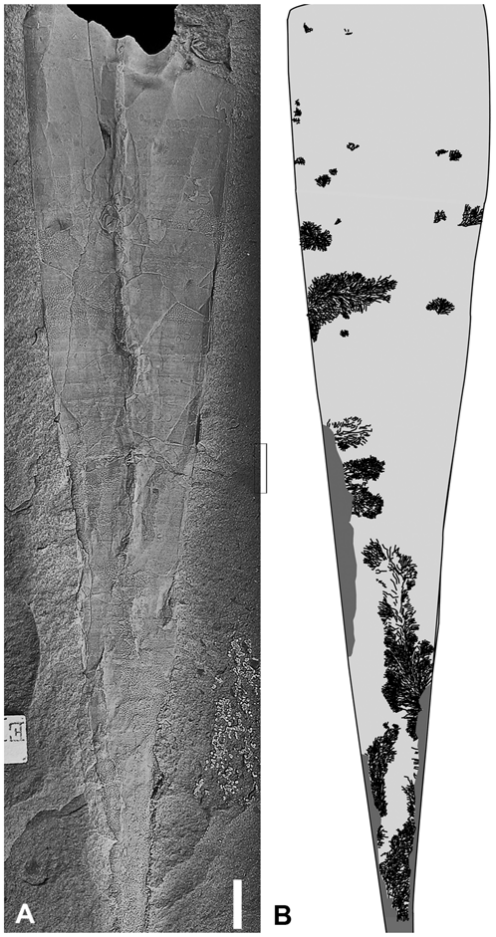
*Isorthoceras* sp. with aligned bryozoan colonization. Specimen NYSM 17627 from Indian Castle Shale, late Katian, New York. Scale bar equals 1 cm.

The conch form is important for the reconstruction of the life habit and habitat of extinct cephalopods. The most abundant cephalopods in Ordovician offshore settings and black shales are relatively small orthoconic longicones with conch lengths of less than 100 mm (Figures 1, 7), intermediate angles of expansion of 5–10° and with a moderate chamber height of less than 0.5 of their diameter (e.g. *Isorthoceras*, *Rioceras*, and various species subsumed under “*Arionoceras*”, and “*Geisonoceras*”). A similar concentration of small orthocerids with moderate chamber spacing is known from the distal, deep shelf environments of the Silurian Ludlow Series, Welsh Borderland, UK [46].

An additional common, and characteristic element in Ordovician offshore settings and black shales are large, slender, orthocones with very low angles of expansion and chamber heights of >0.5 of their diameter (e.g. *Bactroceras*, *Cochlioceras*, and various species subsumed under “*Orthoceras*” and “*Michelinoceras*”). *Sinoceras*, the predominant lituitid in the Pagoda Limestone, is a slender longicone with an apical angle of c. 8°. According to [47] low apical angles of less than 8° are required for deep sea habitats. Only in longicones septa with a spherical cap shape occur, which secure a maximum strength against hydrostatic pressure. Brevicones are restricted by their shell strength to shallow water habitats. Empirical data support the greater conch implosion depths of orthoconic longicones [21], [27], [41], [48].

Many of the predominant orthocones of the Ordovician deep subtidal and black shale environments lacked, or strongly suppressed endosiphuncular and heavy cameral deposits. The life position of these forms can be reconstructed as inclined-vertical, resulting in a poor manoeuvrability and an ability of sluggish forward swimming only [49]. Additionally, the position, and size of the attachment scars of orthoconic longicones indicate small retractor muscles, which were not sufficient for a jet-powered swimming [40]. Consequently, the great majority of cephalopods in Ordovician black shales and distal sediments can be interpreted as sluggish swimming, vertical migrants.

### Taphonomic evidence for pelagic habitats

#### Conch size and fragmentation

Data on shell preservation of the Ordovician deep subtidal and black shale environments are rare. Approximately half of the orthoconic shells in the Utica Shale have the body chamber preserved. In the Utica Shale most orthoconic fragments have a diameter of 5–15 mm (Figure 4) and represent specimens with conch lengths of less than 100 mm. Three of the 98 fragments from the Utica Shale are from putative endocerid shells with diameters of more than 100 mm. These large shells are often heavily fragmented and represent only small parts of the complete conch. The orthoconic fragments in the Fjäcka Shale comprise two distinctive size classes, very small specimens with diameters of less then 5 mm and specimens with diameters of 10–15 mm (Figure 4). The coiled shells are mostly nearly complete, but often the body chambers are broken ventrally and adoral parts are missing. It was mentioned in an earlier investigation that the coiled Utica specimens display a relatively large size compared with that of the Trenton limestone [24, p. 97]. The largest coiled shell in the Utica Shale, a *Trocholites ammonius*, is 78 mm. Seven of the 15 coiled shells of the Utica Shale are larger than 60 mm in diameter. In the Fjäcka Shale samples five specimens of *Discoceras* sp. occur, all with a diameter of >25 mm.

Observations on Recent *Nautilus* [50] and calculations on Paleozoic nautiloids [51] demonstrate the instant chamber refilling and immediate sinking of particularly small and breviconic shells. Additionally, no or little drifting and vertical habitat separation is indicated by taphonomic data and shell implosion pattern in a Silurian “*Orthoceras* limestone” [48]. An instructive additional example is the concentration of “large numbers of *Michelinoceras* sp.” with imploded septa in a hardground layer of the upper Shoemaker beds, early Katian, Tasmania. This hardground bed is interpreted as representing an upwelling zone with an estimated depositional depth of c. 300 m [52, p. 155].

A long and distant *post mortem* dispersal of small shells is therefore highly unlikely. The rarity of brevicones and the lack of small shallow water cephalopods in distal sediments (Figure 3) support these findings and suggest an authochtonous origin of the majority of the small orthoconic shells. In contrast, for the large endocerids and tarphycerids a *post mortem* drifting is probable and an allochthonous origin cannot be excluded. However, a scenario of a general widespread nekroplanktonic cephalopod dispersal, such as postulated by Reyment [53], [54] must be rejected. Reyment's [54] review on cephalopod dispersal neglects the important critical observations and calculations of Westermann, and of Wani et al. [50], [51]. Furthermore, it lacks a discussion of the drastic differences in cephalopod occurrence and preservation pattern between shallow and deep depositional environments.

#### Epizoans

**Figure 8:**
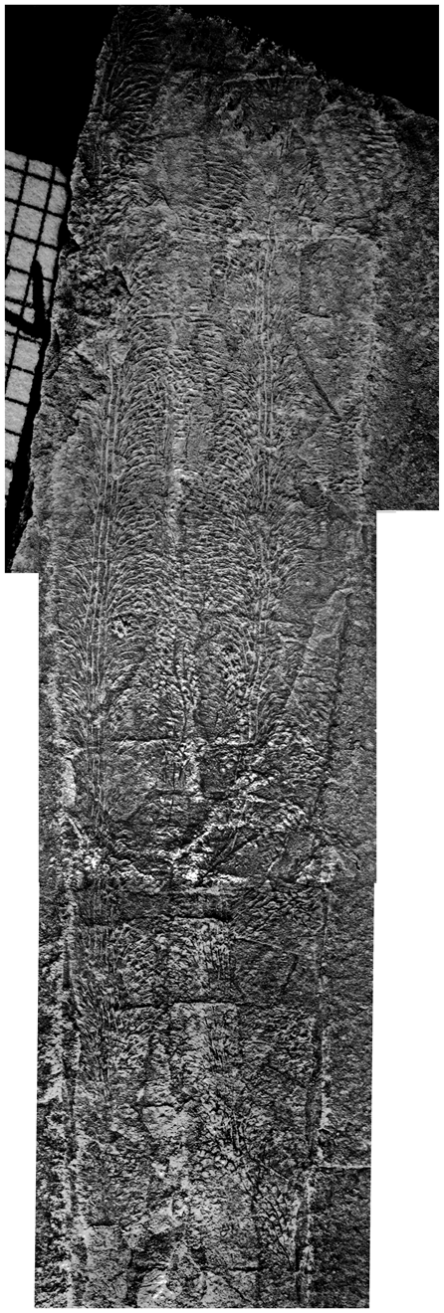
Orthocerida indet. with aligned bryozoan colonization. Specimen PMU 25143 from Fjäcka Shale, late Katian, Dalarna, Sweden. Scale bar equals 1 cm.

Epizoans on cephalopod conchs often occur in Late Ordovician black shale associations [55], [pl. 2. fig. 12, 56]. In the Utica Shale 12% of all cephalopods (n = 98) were colonized by cystosporate bryozoans. Bryozoans commonly occur on the conchs of small orthocones in the Fjäcka Shale. The bryozoans always display an aligned or oriented growth directed toward the conch aperture in the orthoconic specimens (Figures 7, 8). In all cases the apical part of the conch is more heavily overgrown than in adoral sections. No epibionts are known from within the body chamber in conchs of the Fjäcka and Utica Shales. Examples of aligned epibiont growth are known from several Ordovician orthocones [25], [55] and are consistently interpreted as *syn vivo* colonization. In colonized cephalopod specimens the bryozoans grew in a direction concordant with a forward swimming animal with apex up conch position. The lack of epibionts in the body chamber and the apparent lack of *post mortem* overgrowth in the Fjäcka and Utica shales is a strong argument against long drifting periods and of immediate sinking of the shells after death.

**Figure 9:**
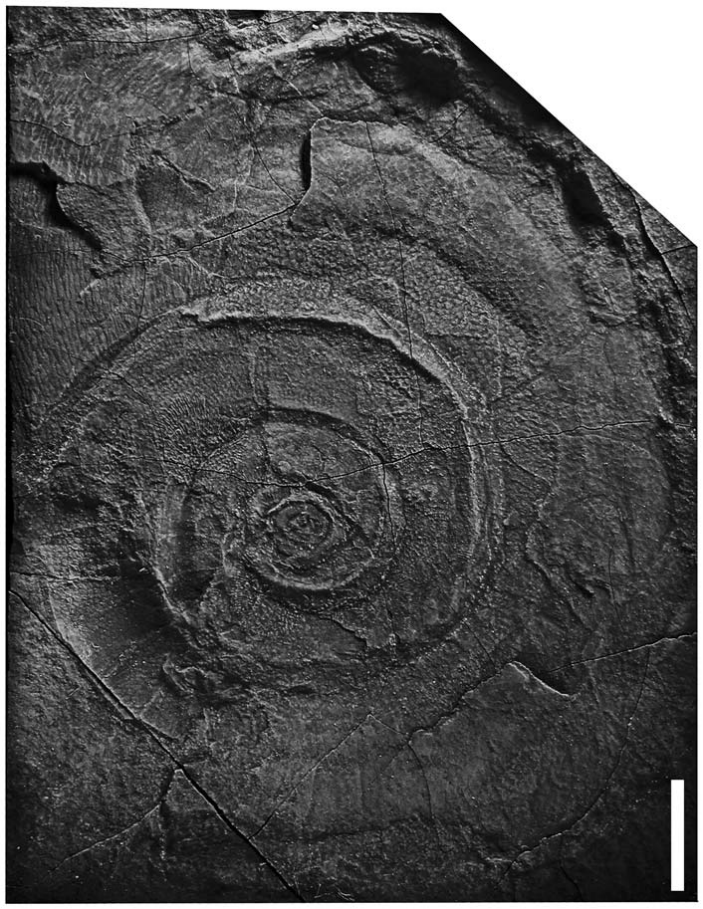
*Trocholites ammonius*, NYSM 9597, from Indian Castle Shale, late Katian, New York with nondirectional bryozoan colonization. Scale bar equals 1 cm.

One coiled specimen with bryozoan overgrowth is known from the Utica Shale [24, p. 97]. Its colonization pattern differs considerably from the orthoconic specimens, it is not directed towards the aperture but grew multi-directionally from several inner whorl loci (Figure 9). A *post mortem* colonization is likely. In the context of the anoxic-dysoxic depositional conditions of the Utica Shale [23] the epibionts of this specimen must be interpreted as a result of a period of drifting of the dead shell.

#### Global paleogeographical ranges

Many of the simple straight orthocones which are common in black shales and distal sedimentary settings are subsumed under *Michelinoceras* (e.g. [52, p. 155]), *Geisonoceras*, and sometimes *Arionoceras* [57], [58]. These characteristic Silurian genera are wastebasket taxa for the often poorly preserved and simple orthocones, which are ubiquitous in black shales and distal settings. The stratigraphic and paleogeographic ranges of these genera are therefore not considered, herein. However, other common taxa were worldwide distributed and characterized by long stratigraphic ranges. *Bactroceras angustisiphonatum*, for example, is known from Australia, Avalonia, Baltica, Laurentia, North, and South China [12], and ranges from the early Floian–Katian [39]. The Darriwilian *Cochlioceras avus* is known from Baltica, the Precordillera terrane, and South China [43]. The genus *Isorthoceras* needs to be revised, but several of its more than ten species potentially can be synonymized. *Isorthoceras* is known from the Darriwilian–late Katian from Avalonia [59], Baltica [60], and Laurentia [25]. Widespread occurrences and long stratigraphic ranges are characteristic and often found in pelagic animals (e.g. [61], [62]).

### Synopsis – Pelagic cephalopods in the Ordovician

The occurrence data show, that cephalopod associations in offshore depositional settings and black shales are characterized by a specific composition. In contrast to shallow depositional environments slender orthocones with thin empty siphuncles are predominant, and breviconic forms are strongly underrepresented. The frequency distribution of conch sizes and the pattern of epibionts indicate an autochthonous origin from the pelagic zone of the majority of the shells. Only for the large shells of endocerids and for coiled forms ambiguous data exist and a *post mortem* drifting is likely. Color marks and the exclusive *syn vivo* epibionts in cephalopods deposited in distal sediments under anoxic–dysoxic and aphotic–dysphotic conditions indicate a pelagic habitat of the predominant taxa. The long stratigraphic ranges and wide paleogeographic distribution are an additional evidence for a pelagic habitat of the common taxa in offshore sediments. The occurrence data are supported by earlier calculations of the mechanical strength against hydrostatic pressure. Slender longicones withstood highest pressures, and therefore were capable of significant vertical migration deep into the mesopelagic zone. The siphuncle shape and structure of Orthocerida and Lituitida was additionally advantageous for a vertical migration. The small spherical apex of orthocerids and lituitids potentially enhanced the buoyancy of the eggs and the early hatchlings and indicates a planktonic early juvenile phase in these cephalopods, similar to bactritoids. A hatching from floating egg-masses, as suggested by [35] for *Bactrites*, is possible.

**Figure 10:**
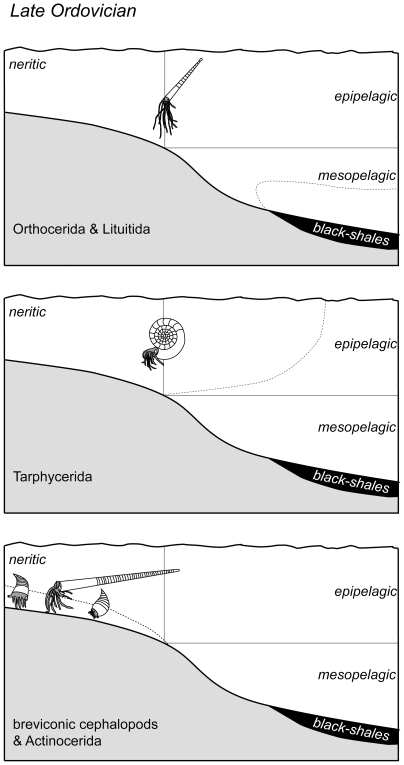
Habitats of selected Late Ordovician cephalopods (dotted lines).

It can be concluded that Orthocerida and Lituitida were slowly swimming vertical migrants of the free water column (Figure 10). The two cephalopod orders are not restricted to open water environments and many taxa must have lived, permanently or during later life phases, in neritic waters, related to the bottom or in reef environments. Orthocerids occur in reefs and other shallow marine settings, they even occur in quartzites and restricted environments of the Middle Ordovician iron ore facies of the Prague Basin [19], [63]. However, we can demonstrate, that orthocerids and lituitids are consistently concentrated in offshore sediments and black shales.

Additionally, endocerids, tarphycerids, and other groups frequently occur in deep offshore settings and black shales. But, it cannot be excluded that these forms are drifted shells. In the Middle Ordovician Elnes Formation, for example, Endocerids are overwhelmingly concentrated in the limestone horizons. The limestones are considered as the shallowest intervals of the formation. No cephalopods occur in depositional depths below the storm wave base, except for very rare, large fragments of endocerids [64]. Rare, large fragments of putative endocerids are also known from the Utica Shale [24]. Large shells of the endocerids and coiled shells have a better potential for drifting than the small orthocerids [50], [51]. The comparatively large size, in some cases the pattern of epibionts of the conch, and the lack of small specimens of endocerids, tarphycerids and other non-orthocerids in the black shales suggest that these occurrences consists of drifted shells. In contrast, the concentration of small, often juvenile orthocerid specimens in the black shales and the consistent pattern of a dominance of orthocerids in offshore environments is clear evidence for the pelagic habitat of these cephalopods.

### Cephalopods and the Ordovician Radiation: Occupation of pelagic habitats

Occurrence data, morphological characters, and taphonomic pattern lead to the conclusion, that Orthocerida and Lituitida are vertical migrants of the free water column, which inhabited, but not exclusively, the open oceans beginning from, at least, the latest Tremadocian. The first occurrences of cephalopods in distal, deep settings are of mid Tremadocian age. But the Tremadocian and Floian occurrences are accompanied by a, sometimes, rich benthos. For many taxa the apex characters are not, and the siphuncular characters are poorly known. Therefore, an epipelagic habitat of Early Ordovician cephalopods cannot be concluded with certainty, and an, at least, intermittent demersal life is likely for these early forms. However, the consistent concentration of Orthocerida and stem group orthocerids in deep subtidal sediments starting from the middle Tremadocian indicates the begin of the occupation of the pelagic zone; i. e. cephalopods, especially adapted for drifting and vertical migrating in the open water, appeared at this time interval in deeper water sediments.

**Figure 11:**
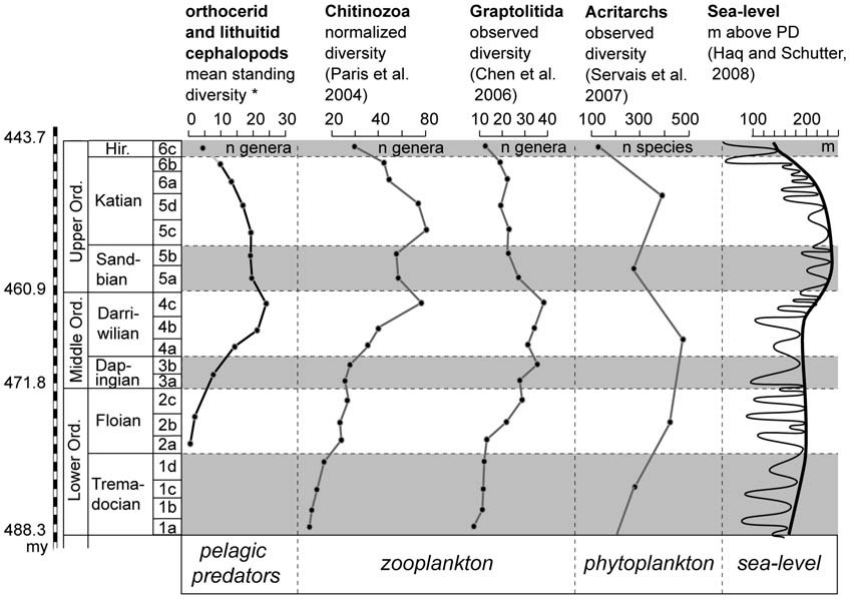
Diversity evolution of Ordovician zooplanktonic organisms compared with global eustatic sea-level curve.

The global diversity trend for orthocerids and lituitids is unique among the Ordovician cephalopods (Figure 11) with an uninterrupted and strong diversification pulse from the Floian to the Darriwilian and a diversity peak in the late Darriwilian (compare [65]). A similar diversification pattern is known from chitinozoans [66], and graptolites [67] (Figure 11). These two zooplanktonic groups show a massive gradual Floian–Darriwilian diversity increase, resulting in a total late Darriwilian diversity peak.

The chitinozoan diversity curves do not differ significantly in disparate paleogeographic regions, with increasing diversities in all palaeocontinents since the Early Ordovician after the first appearance of the group in the early Tremadocian, and it was implied that the main driving factors of the diversification of the group were global [66].

Graptolites were entirely benthic during the Cambrian. The first planktonic graptolites appeared during the Late Cambrian–early Tremadocian bounday interval [67]. The diversity trends of the planktonic graptolites indicate a continuous radiation since the earliest Ordovician up to the late Darriwilian (Figure 11), thus similar to the orthocerid and lithuitid cephalopods and the chitinozoa.

Radiolarians were part of the oceanic plankton at least since the Late Cambrian [68]. Only few data exist for the Ordovician radiolarian diversity, not allowing precise diversity trends, although it appears that a major diversificiation took place during the Early and Middle Ordovician [69], [70]. Occurrences of Cambrian planktonic trilobites are rare [71], [72], but a number of cyclopygid, and telephinid trilobites, and caryocaridids, which are considered as midwater, free swimming arthropods [62], [73] first appeared in the early Tremadocian and provide evidence for the widespread existence of complex pelagic food chains already in the Early Ordovician [62].

Additional data overwhelmingly support the existence of a strong Early Ordovician pulse of the invasion of the open marine realm. Peterson [74] demonstrated the synchronous begin of the exploitation of the pelagic realm by feeding larvae of several independent invertebrate clades during the latest Cambrian to Middle Ordovician. Peterson's [74] data are supported by the appearance of small scaled mollusc larval shells in the fossil record during this time, which are interpreted as a switch to planktotrophy [75]. The appearance of small scaled spherical embryonic shells in cephalopods during the latest Tremadocian is in concordance with this general molluscan trend. The establishment of an open marine food chain, sustainable enough for the development of a diverse fauna of large cephalopod predators was reached early in the Middle Ordovician.

Signor & Vermeji [76] suggested that the repeated and independent invasion of the open water is a result of an escape from an increasing competition and predation pressure at the bottom level. However, Nützel et al. [75] emphasize the synchronous appearance of formerly benthic or benthos-related animals in the open water, the development of planktotrophic larvae and the diversification of suspension feeding organisms. This synchronous appearance likely was not an exclusive result of a benthic predatory escalation, which should be more regionally constrained.

Moreover, the timing and regional pattern of the invasion of the plankton contradicts partially the hypothesis of an escape from the predatory pressure at the bottom level. A predatory escalation at the bottom level is indicated by a diversification of mobile organisms, of predators, and by the increase of rates of bioturbation depths. The major pulse of these processes was during the Middle and early Late Ordovician (see [77]), and the hotspots of diversification were the low latitude carbonate platforms. In contrast the first appearances and the initial diversification of the formerly benthic zooplanktonic organisms date back into the Tremadocian, and pelagic trilobites [73, p. 250], and cephalopods initially diversified in the high latitudes and in peri-Gondwana.

An increased nutrient availability as alternative or additional major cause for the invasion of the open water was proposed [70], [75]. A secular increase in nutrients, or an increase in nutrient availability as an alternative trigger is difficult to demonstrate, and the diversity curves of the fossil phytoplankton [66] are an unreliable measure of the bioproductivity.

The diversity curves of the organic-walled microphytoplankton indicate a strong increase of the number of acritach species and genera since the Late Cambrian (Figure 11). The artificial waste-basked group of the acritarchs most probably includes most of the elements of the organic-walled microphytoplankton of the Palaeozoic oceans (representing phytoplankton groups including prasinophytes, chlorophytes and probably the ancestors of the dinoflagellates). The evolution of the biodiversity of the acritarchs thus most probably represents the evolution of the biodiversity of the organic-walled microphytoplankton. Although it is difficult to relate acritarch diversity with the abundance of organic-walled microphytoplankton in the Palaeozoic oceans, it seems obvious that the rapidly increasing acritarch diversity in the Late Cambrian and Early Ordovician reflects an increasing abundance of organic-walled microphytoplankton at the global scale. It appears thus logical to assume that the presence of important amounts of phytoplankton in the Early Ordovician oceans allowed not only the development of the zooplankton for which the microplankton presented the major food source, but also the development of the planktotrophy, as observed in several clades. The largely parallel diversity trends of the recorded microphytoplankton, the zooplankton and the pelagic cephalopods, which were at the top of the food chain in the Ordovician open water seem to support a link between bioproductivity, and diversification in the water column (Figure 11).

However, an increase in abundance and diversity of the zooplankton must not inevitably be an effect of an increase of nutrient influx or bioproductivity at the base of the food chain. Alternatively, it can be a consequence of a more effective, complex and sustainable food chain or a combined effect of nutrient influx and structure of the food chain [78,79]. During the Late Cambrian–Early Ordovician the pelagic zone was increasingly explored by a diverse and complex zooplankton; radiolarians, graptolites, phyllocarids, trilobites, gastropod larvae, and finally cephalopods, reflect the fossil record of an increasingly complex food chain. It is possible that the increasing complexity and sustainability of the Ordovician pelagic food chain itself was a major factor in driving the diversification.

Additionally, a relation between eustatic sea-levels and marine biodiversity has been discussed in many studies, e.g. [79]. The evolution and diversification of the phytoplankton appears to be related to tectonic (Wilson) cycles of supercontinent rifting and reassembly and associated climate change. A broad correlation between sea-level changes and phytoplankton diversity can thus be observed over the entire Phanerozoic. However, at the scale of our investigation there is no direct relation visible between the diversification of the free swimming animals with the sea level curves, although a general trend of an increasing diversity is paralleled by an early Cambrian to Dapingian global sea-level rise.

At the time it is impossible to explain the patterns of the diversification with simple physical triggers, such as nutrient input or changes in sea level. The search for processes which sufficiently explain the drastic diversification in the Ordovician and the specific role of the plankton evolution is still in its infantry. Our new data add to the impression that the diversification, was a canonical process with a complex temporal pattern in different marine environments, which is poorly explained by isolated triggers. Instead, future research must focus on the Ordovician evolution of the food chain, organismic interaction and on community evolution.

### Conclusion

Cephalopods exclusively inhabited the neritic zone until the earliest Ordovician and entered the pelagic realm during the Tremadocian. Pelagic cephalopods diversified strongly during the late Early Ordovician until the end of the Mid Ordovician, reaching a diversity peak in the Mid Ordovician. The majority of Ordovician pelagic cephalopods were slowly swimming vertical migrants that were physically able to dive into the mesopelagic zone. The first appearance and subsequent diversification of pelagic cephalopods closely followed or was simultaneous to the first appearance of planktotrophic molluscan larvae and common occurrences of trilobites in the pelagic realm. It is difficult to relate the timing and pattern of the expansion of the habitat and the subsequent diversification with possible triggers such as sea-level change and nutrient input. But a relation with the pelagic bioproductivity, and thus with an increasing food availability seems likely. Therewith, future research must focus on processes such as the evolution of the food chain, organismic interaction and community evolution which have the potential to explain the dynamics of the Ordovician Radiation.

## Materials and Methods

The cephalopod occurrences were analyzed using available collections data of the Paleobiology Database (PBDB, http://paleodb.org/cgi-bin/bridge.pl) in february 2009 and from own data ([65], Appendix S1).

The composition and taphonomy of selected black shale cephalopod associations was analyzed on collections from the NRM, NYSM, and PMU.

Institutional abbreviations: NRM-PZ, Naturhistoriska Riksmuseet - Paleozoologi, Stockholm, Sweden; NYSM, New York State Museum, Albany, USA; PMU, Museum of Evolution, University of Uppsala, Sweden.

List of complete names of taxa mentioned in the text: *Ancistroceras* Boll, 1857 [80]; *Arionoceras* Barskov, 1966 [81]; *Bactroceras* Holm, 1899 [82]; *Bactroceras angustisiphonatum* (Rüdiger, 1891) [83]; *Bathmoceras* Barrande, 1867 [84]; *Beloitoceras* Foerste 1924 [85]; *Cochlioceras* Eichwald, 1960 [86]; *Cochlioceras avus* Eichwald, 1860 [86]; *Cyclorangeroceras* Evans 2005 [12]; *Discoceras* Barrande, 1867 [84]; *Eosomichelinoceras* Chen, 1974 Chen, 1974 [21]; *Geisonoceras* Hyatt, 1884 Hyatt, 1884 [87]; *Geisonoceras amplicameratum* (Hall, 1843) [88]; *Isorthoceras* Flower, 1962 [89]; *Isorthoceras romingeri* (Foerste, 1932) new combination [37]; *Isorthoceras tenuitextum* (Hall, 1847) [90]; *Michelinoceras* Foerste, 1932 [37]; *Oncoceras* Hall, 1847 [90]; *Ordogeisonoceras* Frey, 1995 [25]; *Orthoceras* Brugiere 1789 [91]; “*Orthoceras*” *avelinii* (Salter in Murchison, 1859); *Polymeres* Murchison, 1859 [92]; *Sacerdoceras* Evans 2005 [12]; *Semiannuloceras* Evans, 2005 [12]; *Slemmestadoceras attavus* (Brøgger, 1882) [93]; *Sinoceras* Shimizu & Obata, 1935 [94]; *Rioceras* Flower, 1964 [89]; *Trocholites ammonius* Conrad, 1838 [95]; *Tyrioceras* Strand, 1937 [96].

## Supporting Information

Appendix S1 Raw data for analysis of Ordovician cephalopod occurrences.
(0.34 MB XLS)
